# Characterization of Cardiopulmonary Exercise Testing Variables in Patients with Endomyocardial Fibrosis after Endocardial Resection

**DOI:** 10.5935/abc.20170179

**Published:** 2017-12

**Authors:** Ana Luiza C. Sayegh, Marcelo R. dos Santos, Patricia de Oliveira, Fábio Fernandes, Eduardo Rondon, Francis R. de Souza, Vera M. C. Salemi, Maria Janieire de N. N. Alves, Charles Mady

**Affiliations:** Instituto do Coração (InCor) - Faculdade de Medicina da Universidade de São Paulo, São Paulo, SP - Brazil

**Keywords:** Respiratory Function Tests, Exercise Test, Cardiomyopathies, Endocardium / surgery, Cardiomyopathy, Restrictive, Breathing Exercises

## Abstract

**Background:**

Endomyocardial fibrosis (EMF) is a rare disease, characterized by diastolic dysfunction which leads to reduced peak oxygen consumption (VO_2_). Cardiopulmonary exercise testing (CPET) has been proved to be a fundamental tool to identify central and peripheral alterations. However, most studies prioritize peak VO_2_ as the main variable, leaving aside other important CPET variables that can specify the severity of the disease and guide the clinical treatment.

**Objective:**

The aim of this study was to evaluate central and peripheral limitations in symptomatic patients with EMF by different CPET variables.

**Methods:**

Twenty-six EMF patients (functional class III, NYHA) were compared with 15 healthy subjects (HS). Functional capacity was evaluated using CPET and diastolic and systolic functions were evaluated by echocardiography.

**Results:**

Age and gender were similar between EMF patients and HS. Left ventricular ejection fraction was normal in EMF patients, but decreased compared to HS. Peak heart rate, peak workload, peak VO_2_, peak oxygen (O_2_) pulse and peak pulmonary ventilation (V_E_) were decreased in EMF compared to HS. Also, EMF patients showed increased Δ heart rate /Δ oxygen uptake and Δ oxygen uptake /Δ work rate compared to HS.

**Conclusion:**

Determination of the aerobic capacity by noninvasive respiratory gas exchange during incremental exercise provides additional information about the exercise tolerance in patients with EMF. The analysis of different CPET variables is necessary to help us understand more about the central and peripheral alterations cause by both diastolic dysfunction and restrictive pattern.

## Introduction

Endomyocardial fibrosis (EMF) is a neglected disease of unknown cause.^1^ It is characterized by fibrotic thickening of the endocardium and myocardium of one or both ventricles, resulting in ventricular filling restriction,^2^ therefore, is classified as a restrictive cardiomyopathy.^3^

Endocardial scar tissue causes diastolic dysfunction (DD) in these patients.^4^ This diastolic alteration limits ventricular filling and reduces cardiac output (CO).^5^ Previous studies suggested that the major contributor to exercise intolerance in patients with DD is reduced cardiac output.^6,7^ During exercise, this central alteration (decreased CO) that causes DD, is limited by stroke volume, causing early dyspnea and fatigue and, consequently, reduces peak oxygen consumption (VO_2_).^8^

Considering that CO is limited by DD, a stiff left ventricular chamber results in higher filling pressure which usually leads to left atrial dilation causing a pathological hypertrophy.^9^ This pathological alteration is one of the most important adaptations in EMF patients.^10^ However, it has been showed that increased left atrium diastolic volume (LADV) is correlated with low peak VO_2_^11^ in these patients, and that the greater the LADV, the lower is the peak VO_2_.^12^

Currently, for patients with EMF with functional class (FC) III (decompensated) or IV (New York Heart Association, NYHA), the most common treatment is the endocardial resection surgery.^10,13^ However, even after endocardial resection, compensated patients classified between FC I to III, still present reduced peak VO_2_ compared to healthy sedentary subjects.^4^ Haykowsly et al.^14^ demonstrated that the arteriovenous oxygen (A-VO_2_) difference is an independent predictor of reduced VO_2_ from baseline to peak of exercise in patients with DD. Therefore, the authors suggested that this peripheral factor decrease A-VO_2_ difference is one of the most important contributors to exercise intolerance in DD patients. Also, Lele et al.^15^ demonstrated that there is an inverse correlation of left ventricular time to peak filling at peak exercise and peak VO_2_ with peak cardiac output. Thus, changes in left ventricular compliance and relaxation can be more apparent and better understood when exercise is performed.

Cardiopulmonary exercise testing (CPET) has been proved to be a fundamental tool that specifies physical exercise intolerance and has being used as an independent marker of severity and mortality.^16,17^ In this context, CPET has a defined role in the clinical diagnose of exercise intolerance.^12,18^ Peak VO_2_ is a fundamental variable that results from peripheral (A-VO_2_) and central alterations (CO). However, most studies prioritize peak VO_2_ as the main variable, leaving aside other important CPET variables. Workload is a CPET variable that reflexes peripheral limitations once it represents the ability of the muscles to absorb oxygen (O_2_) to produce adequate energy to tolerate the workload during the CPET. Therefore, the higher is the peak workload, the higher is the energy provided by the working muscle. Progressive O_2_ pulse response to incremental exercise is a variable that indirectly represents left ventricular stroke volume (LV-SV) and peripheral extraction of O_2_ per heartbeat. A decreased O_2_ pulse represents an inability to increase LV-SV and maintain CO.^19^ Therefore, CPET variables can help in the identification of different mechanisms of exercise limitation and peripheral mechanisms that can specify the severity of the diseases and guide the clinical treatment. Taking this into consideration, the aim of this study was to evaluate central and peripheral limitations caused by diastolic dysfunction in symptomatic patients with EMF after endomyocardial resection surgery by different CPET variables.

## Methods

### Study population

Fifty-eight patients with EMF attending from the Clinical Unit of Cardiomyopathy at the Heart Institute (Incor), University of Sao Paulo Medical School, Sao Paulo, Brazil were screened for this study. Of those 58 patients, 26 patients met the inclusion criteria. Were also included 15 age-matched healthy sedentary subjects (HS).

Inclusion criteria for patients with EMF were: endocardial resection surgery more than 1 year before the study; functional class III by the NYHA; under optimal treatment (most appropriate medication at the maximum tolerated doses). The inclusion criteria for the HS subjects were: a normal history and physical examinations and; no metabolic, cardiovascular, kidney, and liver diseases.

EMF patients and HS were excluded if they present: regular exercise training activities, history of coronary revascularization or myocardial infarction, diabetes, bi-ventricular pacemakers with or without implantable cardioverter defibrillator and obesity (body mass index, BMI > 30 kg/m^2^).

The investigators were blinded for all measures. The study was approved by the Local Ethics Committee (CAPPesq - number 0130/09) and by the Scientific Research Committee of the Incor (SDC- 3151/08/067). All study participants provided written informed consent. This study was performed according to the declaration of Helsinki and followed the recommendations of the STROBE Statement.^20^

### Echocardiography

Echocardiographic parameters were determined based on the American Society of Echocardiography recommendations as previously described.^21^ EMF assessment was performed through the presence of obliteration in the apex in one or both ventricles, with or without atrioventricular regurgitation.

### Cardiopulmonary exercise test

All patients underwent a maximal progressive exercise test on a cycle ergometer (Ergoline, Spirit 150, Bitz, Germany) to assess maximal oxygen consumption and other ventilatory and cardiovascular parameters, using a ramp protocol with work rate (WR) increments of 5-10 W every minute until exhaustion as described before.^22^ The completion of the test occurred when, despite verbal encouragement, the subject could no longer maintain the exercise and maximal respiratory exchange ratio (RER)^19^ reached > 1.10. Means of gas exchange on a breath-by-breath basis in a computerized system (model Vmax 229, Sensor Medics, Buena Vista, CA) were used to determined pulmonary ventilation (V_E_), VO_2_ and carbon dioxide ventilation (VCO_2_). Anaerobic threshold was estimated as previously described.^23^ Oxygen pulse (O_2_ pulse) was calculated as the ratio between VO_2_ and heart rate (HR) at peak exercise and during CPET.^24^ ΔHR/ΔVO_2_ was measured as the ratio between HR (peak HR - baseline HR) and VO_2_ (peak VO_2_ - baseline VO_2_, beats/L).^23^ ΔVO_2_/ΔWR was evaluated as previously described.^25,26^ We used values of VO_2_ and WR from the 1^st^ minute up to the peak of the exercise.^25^ Ventilatory response (V_E_/VCO_2_ slope) was also calculated as previously described.^25^ We used values of V_E_ and VCO_2_ from the beginning of the CPET up to the peak of exercise.^27^

CPET was assessed in the morning (between 8and 10 a.m.) and all participants were instructed to have the last meal 2 hours before CPET, and to avoid caffeine and high-fat food intake for 24 hours before.

### Statistical analysis

The sample size calculation was based on at least 80% power to detect a mean difference in peak VO_2_ (ml/kg/min) between EMF group and healthy subjects with a 5% significance level. We calculated a total of at least 20 patients with EMF and 15 HS to identify a difference in peak VO_2_.

The Kolmogorov-Smirnov and Levene’s tests were used to assess normality of distribution and homogeneity for each variable. Fisher exact test was used to analyze the distribution of sex. For independent samples, the t-test was used to compare parametric variables, and Mann-Whitney U test was used to compare nonparametric variables. ANOVA for repeated measures and Scheffé’s posthoc test were used to compare the effect of time during CPET on parametric variables, and the Friedman test was used for this same situation for nonparametric variables. Parametric variables were presented as mean ± SD and nonparametric variables were presented as median and interquartile range (IQR, 25%-75%). *P* values < 0.05 were considered statistically significant. All calculations were performed using SPSS software for Windows version 21 (SPSS Inc., Chicago, Illinois, USA).

## Results

### Clinical and physical characteristics

Table 1 shows physical and clinical characteristics. Age, gender and BMI were similar among EMF patients and HS. Functional class, ventricular obliteration, atrial fibrillation and medicaments from EMF patients are displayed in the table.

**Table 1:** Physical and clinical characteristics in patients with endomyocardial fibrosis compared to healthy subjects

Variables	EMF (n = 26)	HS (n = 15)	p value
**Age (years)**	56.9 ± 8.5	53.1 ± 6.1	0.20
**Gender**			
Female	20 (80%)	11 (73%)	0.46
**BMI (kg/m^2^)**	26.9 ± 2.6	27.1 ± 2.2	0.76
**Functional Class (NYHA)**			
II	13 (52%)	-	
III	12 (48%)	-	
**Ventricular obliteration**			
Right	2 (8%)	-	
Left	18 (72%)	-	
Both ventricles	5 (20%)	-	
**Time between surgery and CPET (years)**	6 ± 2	-	
**Atrial fibrilation (n)**	9 (36%)		
**Medications**			
Beta-blokers, n (%)	14 (56%)	-	
ACE/AT1 inhibitors, n (%)	6 (24%)	-	
Diuretics, n (%)	20 (80%)	-	
Digoxin, n (%)	4 (16%)	-	
Espironolactone, n (%)	7 (28%)	-	
Statins, n (%)	10 (40%)	-	
Anticoagulants, n (%)	5 (20%)	-	
Antiarrhythmic, n (%)	4 (16%)	-	

Parametric variables are presented as mean ± SD. EMF: endomyocardial fibrosis; HS: healthy subjects; n: number; BMI: body mass index; LV: left ventricular; ACE: angiotensin converting enzyme; AT1: angiotensin II receptors type I.

### Echocardiographic parameters

Echocardiographic variables are shown in Table 2. Although left ventricular ejection fraction (LVEF) was normal in EMF patients, it was decreased compared to HS. Left ventricular end-diastolic volume (LV-EDV), left ventricular end-systolic volume (LV-ESV) and LADV were increased in EMF. On the other hand, LV-SV was similar in both groups.

**Table 2:** Echocardiographic variables in patients with endomyocardial fibrosis compared to healthy subjects

Variables	EMF (n = 26)	HS (n = 15)	p value
LVEF (%)	56 ± 8	63 ± 4	0.01
LV-EDV (mL)	83,1 (66.5-169.7)	57.0 (51.3-96.0)	0.04
LV-ESV (mL)	35.8 (26.4-82.6)	22.5 (20.0-32.3)	0.03
LV-SV (mL)	48.3 (37.3-76.7)	34.5 (32.3-65.3)	0.09
LADV (mL)	47.7 (36.3-73.4)	34.0 (26.0-43-0)	0.04

Parametric variables are presented as mean ± SD and nonparametric variables are presented as median and interquartile range (IQR, 25%-75%). EMF: endomyocardial fibrosis; HS: healthy subjects; n: number; LVEF: left ventricular ejection fraction; LV-EDV: left ventricular end-diastolic volume; LV-ESV: left ventricular end-systolic volume; LV-SV: left ventricular stroke volume; LADV: left atrial diastolic volume.

### Cardiac function, hemodynamic parameters and functional capacity

CPET variables are displayed in Table 3. Rest HR, peak end-tidal partial pressure for CO_2_ (PetCO_2_), VE/VCO_2_ slope and RER were similar between the groups. Peak HR, peak workload, peak VO_2_, peak O_2_ pulse, peak VCO_2_ and peak V_E_ were decreased in EMF compared to HS. Also, ΔHR/ΔVO_2_ and ΔVO_2_/ΔWR were increased in EMF patients compared to HS.

**Table 3:** Maximal cardiopulmonary exercise test in patients with endomyocardial fibrosis compared to healthy subjects

Variables	EMF (n = 26)	HS (n = 15)	p value
Rest HR (bpm)	69 (61-75)	77 (73-86)	0.01
Peak HR (bpm)	126 ± 18	164 ± 18	< 0.0001
Peak workload (watts)	55 (45-78)	150 (110-180)	< 0.0001
Peak VO_2_ (ml/kg/min)	16.2 ± 3.1	24.5 ± 4.6	< 0.0001
Peak VO_2_ (L/min)	1.106 ± 0.274	1.800 ± 0.389	< 0.0001
Peak O_2_ pulse (ml/beats)	8.8 (7.3-10.0)	10.5 (8.8-13.0)	0.03
Peak VCO_2_ (L/min)	1.206 ± 0.280	2.105 ± 0.431	< 0.0001
Peak PetCO_2_ (mmHg)	31 ± 5	35 ± 5	0.18
Peak V_E_ (L/min)	41 (37-55)	68 (53-83)	< 0.0001
ΔHR/ΔVO_2_ (beats/L)	72 ± 25	56 ± 17	0.04
ΔVO_2_/ΔWR (ml/min/W)	12.5 ± 0.3	10.0 ± 0.1	< 0.0001
V_E_/VCO_2_ slope	34 (29-36)	29 (26-34)	0.12
RER	1.12 ± 0.11	1.16 ±0.06	0.18

Parametric variables are presented as mean ± SD and nonparametric variables are presented as median and interquartile range (IQR, 25%-75%). EMF: endomyocardial fibrosis; HS: healthy subjects; n: number; HR: heart rate; VO_2_: oxygen consumption; VCO_2_: carbon dioxide ventilation; PetCO_2_: end-tidal carbon dioxide; V_E_: pulmonary ventilation; O_2_: oxygen; RER: respiratory exchange ratio.

**Figure 1:**
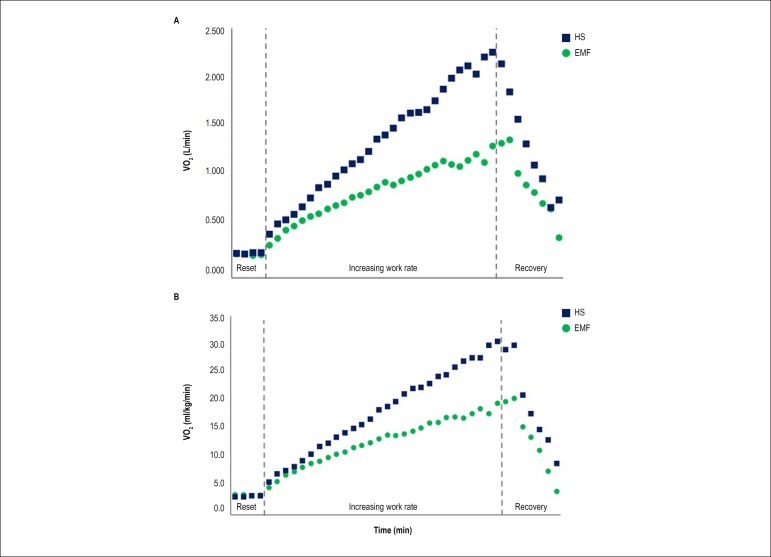
Representative peak VO2 response during exercise in one endomyocardial fibrose patient and one healthy subjects. A) Peak VO2 in absolute units; B) Peak VO2 in relative units. EMF: endomyocardial fibrose; HS: healthy subject; VO2: oxygen consumption.

**Figure 2:**
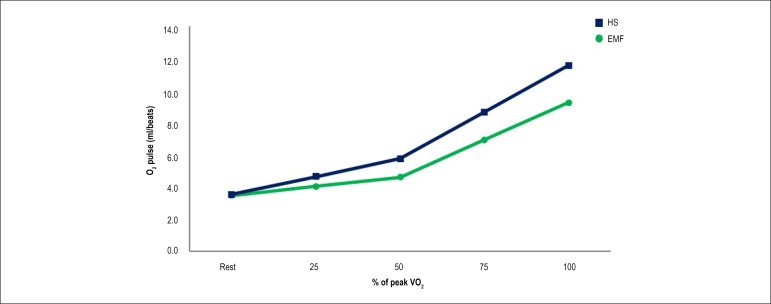
Representative progressive O2 pulse response to incremental exercise in one endomyocardial fibrose patient and one healthy subject. EMF: endomyocardial fibroses; HS: healthy subjects; O2: oxygen; VO2: oxygen consumption.

**Figure 3:**
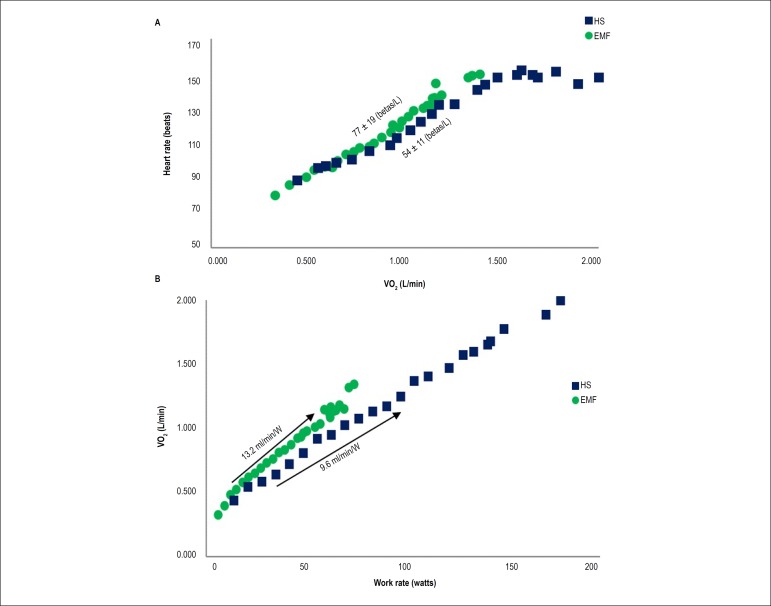
Representative ΔHR/ΔVO2 and ΔVO2/ΔWR in one endomyocardial fibrose patient and one healthy subject. A) ΔHR/ΔVO2 in one endomyocardial fibrose patient compared to one healthy subject. B) ΔVO2/ΔWR in one endomyocardial fibrose patient compared to one healthy subject. EMF: endomyocardial fibroses; HS: healthy subjects; HR: heart rate; VO2: oxygen consumption.

Figure 1(A and B) is a representative of VO_2_ response during exercise (absolute units and relative units, respectively) in one patient with EMF and in one HS. Figure 2 represents the progressive O_2_ pulse response to incremental exercise in one subject from each group (EMF and HS). Figure 3(A and B) shows the increased ∆HR/∆VO_2_ and ∆VO_2_/∆WR (respectively) in one patient with EMF and in one HS.

## Discussion

We know that peak VO_2_ is one of the most important variables to describe exercise tolerance in humans. However, other parameters of CPET can provide additional information on the exercise capacity. The aim of this study was to evaluate different CPET parameters that could help us understand the physical limitations caused by DD in symptomatic patients with EMF. We demonstrated that CPET variables were impaired in symptomatic EMF compared to HS. These findings show that exercise intolerance in these patients is caused by central and peripheral alterations of the restrictive cardiomyopathy condition.

The fibrotic tissue in the ventricles and in the papillary muscles provokes filling restriction, and this alteration causes severe hemodynamic disturbances. Even knowing that ejection fraction is normal or slightly reduced, the stroke volume in EMF patients is decreased, leading to poor peripheral perfusion.^28^

In patients with heart failure and systolic dysfunction, the importance of functional capacity is well described.^29,30^ Reduced peak VO_2_ is correlated with increased hospitalization and mortality rate.^31^ In patients with EMF, peak VO_2_ reduction can be related to 1) a fixed LV-EDV that affects LV-SV increases during a maximal cardiac work, and thereby affects CO increases; 2) blunted maximal workload during maximal exercise testing, showing that these patients cannot handle a high workload due to an inefficient cardiac work; 3) the difficulty in increasing the CO during maximal exercise test can provoke a reduction in peripheral blood flow favoring an early fatigue. It is important to highlight that all these patients had been submitted to endocardial resection surgery, and despite the procedure, they still presented inefficient O_2_ distribution and consumption compared to HS.

Other interesting CPET variable explored in this study was the increased ΔVO_2_/ΔWR in patients with EMF. This variable evaluates: 1) the metabolically induced vasodilation and thus the increased O_2_ flow to the place of demand; 2) an increased O_2_ uptake when transforming lactate to glycogen by tissues actively involved in gluconeogenesis; and 3) an increased O_2_ demand of breathing muscle.^32^ Therefore, this variable represents the importance of peripheral metabolism during incremental exercise. Knowing that VO_2_ increases progressively and linearly to increases in workload during CPET, healthy sedentary subjects consume a constant O_2_ amount to produce energy and fulfill the metabolic demands during a specific work. Regardless of age or physical training level, the normal value for HS is 10 mL/min/watts.^33^ In this study, we showed that EMF patients have a ΔVO_2_/ΔWR of 12.5 ml/min/watts. Change in ∆VO_2_/∆WR reflects a low A-VO_2_ difference^34^ that may contribute significantly to exercise limitation in EMF patients. Also, the ∆VO_2_/∆WR impairment may be explained by abnormalities of oxygen extraction in skeletal muscle or conditions causing reduced blood flow to exercising muscle. Abnormal skeletal muscle fibers with low mitochondrial density are associated with reduced oxidative capacity due to reduced oxygen use and inappropriate vascular responses to exercise.

On the other hand, to evaluate central limitations during CPET, we analyzed O_2_ pulse. This variable is calculated by the ratio between VO_2_ and HR,^24^ and consequently, it can be used as a noninvasive indicator of stroke volume.^35^ It normally rises progressively throughout exercise; however, a decreased value suggests decreasing stroke volume during exercise.^36^ EMF patients show a reduced O_2_ pulse which could be explained, at least in part, by a difficulty in increasing CO via systolic volume caused by fixed diastolic volume. In consequence, the increase in CO during CPET is highly dependent on increases in HR, limiting the increase in O_2_ pulse.

Another important variable that can be used to evaluate central alterations is the ΔHR/ΔVO_2_ ratio. It indicates the necessary cardiac work to provide 1 liter of O_2_ to fulfill metabolic demands, such as muscle energy for a specific workload.^23^ Reduced values of ΔHR/ΔVO_2_ in EMF patients, even after endocardium resection, demonstrate that these patients have an increased cardiac work to consume the same amount of O_2_ compared to HS. As previously stated by Ramos et al.,^23^ a reduced stroke volume and/or diminished A-VO_2_ difference would lead to a steeper ΔHR/ΔVO_2_, whereas cardiac dysfunction, decreased arterial O_2_, and impaired muscle aerobic capacity can increase ΔHR/ΔVO_2_.

Finally, this study demonstrated that patients with EMF have an impaired cardiac function and peripheral alterations that influence the exercise intolerance. Taking all into consideration, we demonstrated the importance of the combined evaluation of different CPET variables. All these variables together may be an important key to evaluate patients with restrictive cardiomyopathy due to EMF.

### Limitations

There are limitations in this study. EMF is a rare and neglected disease, and for this reason we studied a small sample size. We only studied patients with EMF, which is the most common etiology of restrictive cardiomyopathy in tropical countries. Therefore, we cannot assume that these results will be found in other forms of restrictive cardiomyopathy or diastolic dysfunction. All patients from this study had been submitted to resectional surgical for fibrosis; therefore, we do not know whether similar results would be found in patients before the surgical procedure. Lastly, central and peripheral CPET variables were evaluated noninvasively, and thereby in an indirect way. It would be of great interest to reproduce this study evaluating cardiac output and A-VO_2_ difference in a direct way.

## Conclusion

Determination of patient’s aerobic capacity by noninvasive respiratory gas exchange during incremental exercise provides additional information about the exercise tolerance in patients with EMF. The analysis of different CPET variables is necessary to help us understand more about the central and peripheral alterations caused by both diastolic dysfunction and restrictive pattern.
